# Transient traumatic isolated neurogenic ptosis after a mild head trauma: a case report

**DOI:** 10.1186/s12886-015-0153-5

**Published:** 2015-11-08

**Authors:** Guichen Li, Yang Zhang, Xiaobo Zhu, Kun Hou

**Affiliations:** Department of Neurology, The First Hospital of Jilin University Changchun, Changchun, Jilin China; Department of Neurosurgery, The First Hospital of Jilin University Changchun, Changchun, Jilin 130021 China

**Keywords:** Transient traumatic isolated neurogenic ptosis (TTINP), Head trauma, Oculomotor nerve palsy

## Abstract

**Background:**

Transient traumatic isolated neurogenic ptosis (TTINP) is a sporadically reported rare entity. However, to the best of our knowledge, nearly all the reported cases are either secondary to direct periorbital trauma or surgery. We would like to report on a case of TTINP with countre-coup injury of the periorbital region.

**Case presentation:**

A 49-year-old female slipped and fell down while walking. She was hospitalized with a moderate headache and undisturbed mental state. The patient recalled that the force bearing point was her occipital region. Physical examination and computed tomography (CT) on admission showed right isolated ptosis and mild contusion and laceration in the bilateral frontal cortex. Further radiological investigation revealed nothing remarkable except for a fracture of the superior portion of the right medial orbital wall. She was managed conservatively and recovered completely in two months.

**Conclusion:**

TTINP might manifest as a unique entity with a relatively mild, reversible, and non-devastating injury to the terminal branch of the oculomotor nerve and for which perhaps no special treatment is needed. The proposed mechanism is injury of the terminal branch of the superior division of the oculomotor nerve.

## Background

Isolated ptosis is a manifestation of various clinical conditions. It may be congenital, acquired, myogenic, neurogenic, mechanical, aponeurotic, or traumatic in nature [1]. However, transient traumatic isolated neurogenic ptosis (TTINP) is rather rarely reported in the literature [2, 3]. To the best of our knowledge, nearly all the patients reported with TTINP have histories of direct injury to the periorbital region [2–4]. In this report, a case of TTINP secondary to countre-coup injury to the periorbital region was discussed.

## Case presentation

**Fig. 1:**
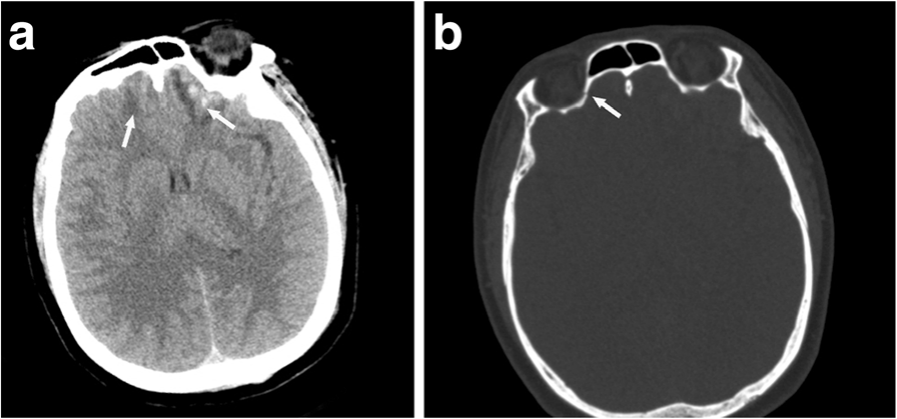
A computed tomography scan revealed a mild bilateral contusion and laceration in the frontal poles (**a**) and a fracture of the superior portion of the right medial orbital wall (*arrow head*) (**b**)

**Fig. 2:**
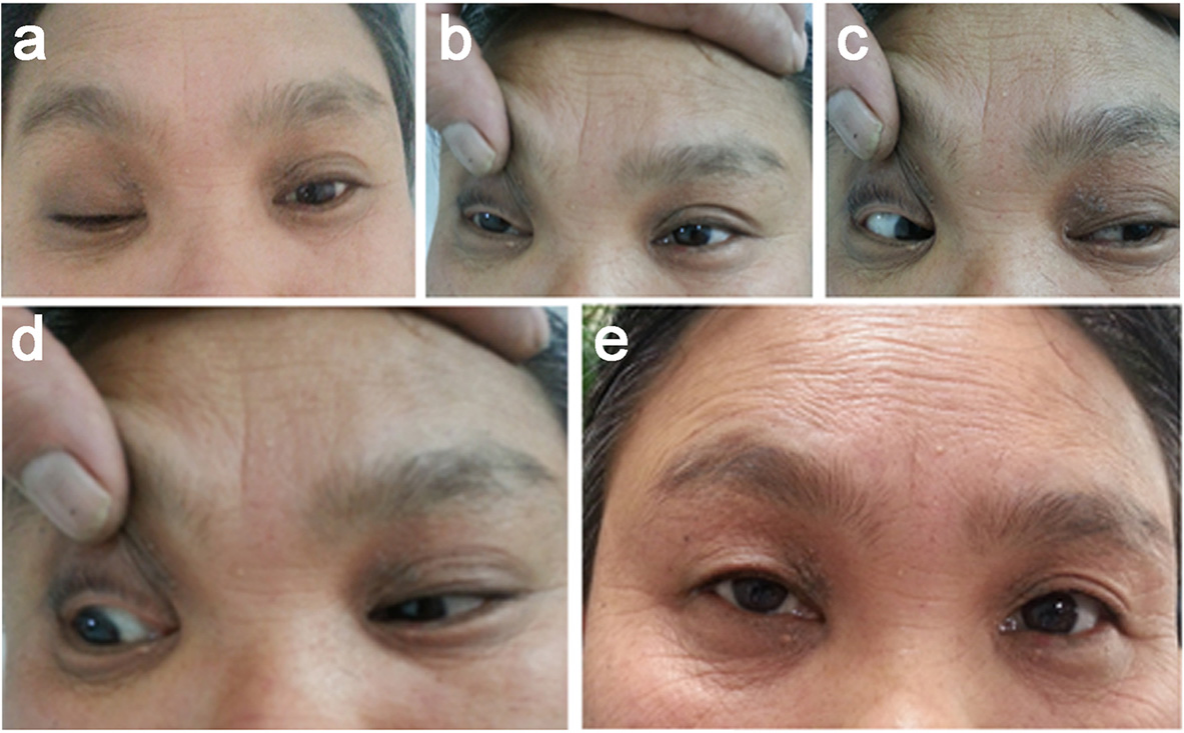
Physical examination at two days after admission showed right ptosis with the bruised bilateral upper eyelids (**a**); right eye in primary position (**b**); normal extraocular motility of the right eye in right and left gaze (**c-d**); the right levator function was the same as the left-side except an indistinct upper eyelid crease in the fourth week after the accident (**e**)

A 49-year-old female slipped and fell down while walking. Two hours later, the patient was hospitalized (The First Hospital of Jilin University, Changchun, China) with a moderate headache. A computed tomography (CT) (Fig. 1a) performed on admission showed a mild contusion and laceration in the bilateral frontal cortex. The patient was undisturbed in mental state through the accident and recalled that the force bearing point was her occipital region. Physical examination results were unremarkable except a right complete ptosis and swelling and contusion of the occipital region. The patients’ bilateral upper eyelids were gradually bruised in two days after the fall (Fig. 2a). The patients’ Glasgow Coma Scale score was 15. She denied alteration of her vision. The ophthalmic examination showed normal extraocular motility and pupillary responses without anisocoria (Fig. 2b-d). High-resolution CT images showed a fracture of the superior portion of the right medial orbital wall (Fig. 1b). Further magnetic resonance imaging and CT angiography revealed no other intracranial or intraorbital lesion that may be responsible for her ptosis. The patient was administered Haemocoagulase Agkistrodon (KONRUNS Pharmaceutical Co., Ltd., Beijing, China) intravenously for hemostasis in the initial three days (2U once daily) and Deproteinized Calf Blood Extractives (Harbin Sanctity Pharmaceutical Co., Ltd., China) (1.2 g once daily) intravenously for neuroprotection for 14 days. Some unspecified analgesic medications were also administered intermittently. Fourteen days later, the patient was discharged from the hospital with obvious remission of headache. However, the right ptosis persisted with levator function of 5 mm (left levator function was 14 mm). During outpatient follow-up, it was found that the right levator function was the same as the left-side except an indistinct upper eyelid crease in the fourth week after the accident (Fig. 2e). The patient’s eyelid crease became normal at two months after the hospital discharge. One year follow-up showed no recurrence of eyelid dysfunction.

## Conclusions

Although there is no official definition of TTINP, according to the literature, TTINP has at least two identical characteristics: a) TTINP occurs secondary to direct or indirect injury of the upper eyelid and its innervation; b) self-limited with recovery after a certain period of time with conservative treatments or just observation [2, 5, 6]. There are also several reports of isolated ptosis after medial wall reconstruction of the orbit [5, 6]. However, nearly all the reported cases are either secondary to direct periorbital trauma or surgery [2, 3, 5, 6]. To the best our knowledge, this is the first report of TTINP with no direct trauma of the periorbital region.

Based on the etiology, ptosis may be myogenic, aponeurotic, or neurogenic [1]. TTINP could be classified as neurogenic which has no devastating injuries to the levator palpebrae superioris (LPS) muscle. The LPS muscle is innervated by a terminal branch from the underlying superior rectus muscle which receives nerve control from the oculomotor nerve. TTINP may be caused by injury of the terminal branch of the superior division of the oculomotor nerve. In this case report, TTINP was not secondary to direct injury of the upper eyelid and it recovered spontaneously. A fall onto the back of the head may lead to a secondary fracture of the bones of the orbit, known as a countre-coup injury. The frontal lobes of the brain may also be damaged, as described in the present patient. Subsequently, entrance of blood into the orbit due to the crack in the walls of the orbit often appears as bruising of the eyelids. The terminal branch of the superior division of the oculomotor nerve was stretched and shocked during this process.

The management of TTINP differs in different reports, ranging from simple treatments of the primary diseases to corticosteroid administrations [2, 3, 5]. However, there is no difference in the duration of recovery process, which ranges from two weeks to six months regardless of chosen treatment options in reported literature cases [2, 3, 5, 6]. Although previous reports have shown that some patients with complete oculomotor nerve palsy benefit from some invasive managements, TTINP might manifest as a unique entity with a relatively mild, reversible, and non-devastating injury to the terminal branch of nerve and for which perhaps no special treatment is needed [7]. Corticosteroid treatment should be avoided, if not needed, in order to prevent undesired side effects. In this report, the patient spontaneously recovered in two months just with conservative treatment. However, given the small number of reported cases, no conclusion regarding the best therapy could be derived from the existing literature till now. Moreover, the management of TTINP should be based on the future accumulation of reports of cases and case series.

### Consent

Written informed consent was obtained from the patient for publication of this Case report and any accompanying images. A copy of the written consent is available for review by the Editor of this journal.

## Abbreviations

CT: computed tomography
LPS: levator palpebrae superioris
TTINP: transient traumatic isolated neurogenic ptosis
